# 

**Published:** 1920-02-14

**Authors:** 


### Wills of Medical Men.

Dr. Joseph Quirke, of Endwood Court, Handsworth, left estate valued at £62,376.

Dr. C. H. Carter, of Bromley, late of Great Cumberland Place, W., at one time physician-accoucheur at St. George's Dispensary, and consulting physician to the Hospital for Women, Soho Square, left £22,988.

Mr. W. K. Walls, of Withington, Manchester, hon. surgeon to the Amalgamated Hospitals of St. Mary and the "Southern," left estate valued at £18,382.

Professor Frederick Page, F.R.C.S., M.D., of Newcastle-on-Tyne, at one time Registrar of the Durham University College of Medicine, Lecturer on Medical Jurisprudence, and President of the College, left £16,732.

Captain J. C. Simpson, R.A.M.C., M.D., of Cambridge, formerly house physician to the Royal Infirmary, Edinburgh, left £7,000, on the death of the survivor of his wife and sister, to the Royal Medical Benevolent Fund and the Indigent Blind Visiting Society. His will was proved at £8,378.

### THE COLLEGE OF NURSING (Limited by Guarantee).

LONDON CENTRE.

GENERAL MEETING.

A GENERAL MEETING of members of the London Centre will be held in the Board Room, Middlesex Hospital, Mortimer Street, W. 1 (by kind permission of the Weekly Board) on Monday, February 16, at 7 P.M.

*DAILY TELEGRAPH* SHILLING FUND.

In connection with the *Daily Telegraph* Shilling Appeal for the Nation's Fund for Nurses, February 16 has been chosen for the Hospital Nurses' Day, when the contributions of nurses in groups or singly will be specially welcomed. Members of the London Centre are asked to help, and to get their friends to do the same. Contributions may be sent to Miss Copeman, Treasurer of the London Centre, Paddington Military Hospital, Harrow Road, who will forward the total to the *Daily Telegraph* as from "Members of the London Centre of the College of Nursing." No individual receipts will be sent out. The total will be announced in these columns in our issue of February 21.

EDINBURGH CENTRE.

LECTURES.

WEDNESDAY, FEBRUARY 25.—James Burnet, M.A., M.D., F.R.C.P.E., "Influenza."

THURSDAY, MARCH 25.—A. Logan Turner, M.D., F.R.C.S.E., "The Nose, Ear, and Throat." A lantern demonstration illustrating the anatomy of the regions and some of the common ailments.

THURSDAY, APRIL 29.—W. G. Sym, M.D., F.R.C.S.E., "Eye Diseases from a Nursing Point of View."

These lectures will be given in the Nurses' Club, 8 Drumsheugh Gardens, Edinburgh. Time will be announced later.

### MATRONS' AND SISTERS' APPOINTMENTS.

TOXTETH PARK INFIRMARY, LIVERPOOL.—Miss Fannie Brotherton has been appointed matron and superintendent nurse. She was trained at Toxteth Park Poor-law Infirmary, and was subsequently appointed staff nurse at Chelsea Poor Law Infirmary, charge nurse at Prescot Union Infirmary, head night nurse at Aston Union Infirmary, and superintendent nurse successively at Barnsley, Belper, and Sculcoates Union Infirmaries.

GENERAL HOSPITAL, ALTRINCHAM.—Miss Lornetta Roskell has been appointed night sister. She was trained at the above hospital, and has held appointments in war hospitals.

MATERNITY HOSPITALS AND CHILD WELFARE CENTRE, CARLISLE.—Miss Gertrude Monk has been appointed matron. She was trained at the Royal Portsmouth Hospital, and has since been night sister at the Royal Southern Hospital, Liverpool, and at the North Lonsdale Hospital, Barrow-in-Furness, and sister at Queen Charlotte's Hospital.

PRESTON, FULWOOD, AND LONGRIDGE JOINT HOSPITAL.—Miss Minnie Riley has been appointed matron. She was trained at Birmingham Union Infirmary, has been sister and deputy matron of the Isolation Hospital, Horwich, and matron of the Isolation Hospital, Ormskirk.

